# The Informational Substrate of Chemical Evolution: Implications for Abiogenesis

**DOI:** 10.3390/life9030066

**Published:** 2019-08-08

**Authors:** Andrés de la Escosura

**Affiliations:** 1 Department of Organic Chemistry, Universidad Autónoma of Madrid, Cantoblanco Campus, 28049 Madrid, Spain; andres.delaescosura@uam.es; 2 Department of Organic Chemistry, Institute for Advanced Research in Chemistry (IAdChem), Cantoblanco Campus, 28049 Madrid, Spain

**Keywords:** chemical evolution, prebiotic systems chemistry, autonomous chemical systems, information processing, chemosemiosis

## Abstract

A key aspect of biological evolution is the capacity of living systems to process information, coded in deoxyribonucleic acid (DNA), and used to direct how the cell works. The overall picture that emerges today from fields such as developmental, synthetic, and systems biology indicates that information processing in cells occurs through a hierarchy of genes regulating the activity of other genes through complex metabolic networks. There is an implicit semiotic character in this way of dealing with information, based on functional molecules that act as signs to achieve self-regulation of the whole network. In contrast to cells, chemical systems are not thought of being able to process information, yet they must have preceded biological organisms, and evolved into them. Hence, there must have been prebiotic molecular assemblies that could somehow process information, in order to regulate their own constituent reactions and supramolecular organization processes. The purpose of this essay is then to reflect about the distinctive features of information in living and non-living matter, and on how the capacity of biological organisms for information processing was possibly rooted in a particular type of chemical systems (here referred to as autonomous chemical systems), which could self-sustain and reproduce through organizational closure of their molecular building blocks.

## 1. Introduction: Stating the Problem

### 1.1. Chemical Evolution

In the context of origin-of-life research, the concept of *chemical evolution* is central, as it encompasses plausible physicochemical mechanisms by which the first living protocells could have been assembled. Historically, the term chemical evolution began to be used shortly after the first steps in the field of prebiotic chemistry were taken [1], yet with a loose meaning. Its use has gained a renewed energy in recent years, thanks to the emergence of systems chemistry [2,3]. In this research community there is a general view that, in order to understand the transition from inanimate matter to living organisms, complexity must be embraced at the chemical level. According to the current consensus, the first living protocells must have comprised, at least (i) a protocellular compartment, (ii) a protogenome, and (iii) an autocatalytic metabolic network supporting the system with energy and substrate molecules [2]. Moreover, the replication dynamics of the three subsystems must have been coupled for the efficient reproduction of the system as a whole. The problem is that these requirements involve a great level of complexity, regarding both the molecular structure of the protocell components and their dynamics of interaction, whose establishment seems highly unlikely in the absence of an evolutionary driving force. 

Indeed, some researchers have postulated that in populations of self-replicating molecules, or in collectively autocatalytic networks, small variations in the kinetics of their constituent reactions can lead to evolutionary dynamics, including processes such as mutation, selection, and cooperation [4,5,6]. This has contributed to making chemical evolution a key concept for tackling the abiogenesis problem [7,8,9]. However, it disregards some basic functions that would be crucial to implement chemical systems with a potential to self-sustain and evolve. Such functions include the capacity to maintain the system in an out-of-equilibrium state [10,11], or the necessary separation of the system from the environment through a permeable boundary [12]. The necessity of these functions, together with other thermodynamic and kinetic requirements, implies an internal organization that goes beyond the mere existence of replicating entities. In other words, chemical replicators (i.e., molecular species that make copies of themselves through autocatalysis) must be compartmentalized and supported by a protometabolism. Otherwise, the replicating entities, no matter what kind of molecule they are based on (e.g., oligonucleotides, peptides, synthetic molecules, etc.), would be subject to dilution, degradation, or side reactions, and just decay into thermodynamic sinks. 

An important line of experimental work, focused on the construction of protocellular assemblies, actually aims at integrating the three basic subsystems through different kinds of physicochemical processes [13,14,15,16,17]. However, research on self-replication, autocatalytic networks or self-reproducing compartments faces several inherent problems [18]. Most of that work has been performed with molecular components taken from existing living organisms (e.g., phospholipids, peptides, oligonucleotides, etc.), assuming that they would have been available on prebiotic Earth. This is a useful approach to study functional models of the first protocells, yet it is highly improbable that those biomolecules could have been produced spontaneously through random processes (condensation reactions, amino acid, or nucleotide polymerizations, respectively), in sufficient quantities and with adequate structure/sequence to exert their role. Moreover, a strong limitation of these approaches lies in the difficulty of integrating the complex dynamic behaviors of each separate subsystem. In order to overcome these limitations, chemical evolution must have required, from the beginning, molecular assemblies that were able to regulate the production of their own ingredients from the simplest building blocks (e.g., fatty acids, simple sugars, amino acids, nucleobases, etc.).

### 1.2. Evolution of Autonomous Chemical Systems (ACSs)

With the purpose of gaining deeper insights about the mechanisms of chemical evolution, two colleagues and I recently proposed an extension of the Darwinian framework to the study of autonomous molecular assemblies [19]. A fundamental question in this respect is how simple a chemical system with evolutionary capacities could possibly be. In order to be useful, this conceptual framework considers complexity from a functional point of view, instead of a structural one. The notion of *function* in this context is understood as in physiology, i.e., as any specific contribution by a distinct part of a system to the maintenance of the system as a whole [20]. Yet, projection of this notion of function to a chemical scenario is required if one aims to explain how the first cells emerged from non-living matter. In order to do so, we have suggested that the study of chemical evolution must shift its attention from populations of naked molecular replicators to populations of heterogeneous, compartmentalized and functionally integrated assemblies of molecules. The latter could be a useful, sufficiently broad working definition of a protocell, encompassing not only the normally accepted model (a lipid vesicle containing an RNA replicator and the network of reactions supporting their replication processes), but also the first evolutionary chemical systems composed of much simpler building blocks. These building blocks should actually be simple enough to be obtained and accumulated through regular prebiotic synthetic pathways, such as the examples shown in Table 1. 

The above conceptual framework is the outline of a theory of autonomous chemical systems (ACSs) [19], i.e., chemical systems which self-sustain and reproduce by their own, marking the minimal set of functions that they must present in order to engage in an evolutionary process. It could also serve to evaluate the level of complexity achieved by current prebiotic chemistry, and to assess the degree of aliveness of future bottom-up protocells. This would be done by contrasting how the molecular mechanisms of a protocell ensure kinetic control (e.g., coordinating through catalysis the different reactions in time), spatial control (e.g., providing a semipermeable physical separation from the environment, which preserves minimal concentration thresholds of the relevant molecular species), thermodynamic control (e.g., favoring key reactions that are energetically disfavored), and variability control (e.g., preserving the main protocell features through its evolutionary process). Establishing these four types of control mechanisms could enable *organizational closure*, i.e., that the set of system’s components and their physicochemical relationships self-sustain, which is key to unify them and let the system be autonomous [21,22]. However, the abstract nature of such a framework represents a strong limitation for it to guide the lab implementation of ACSs. One of the purposes of this essay is to address the physicochemical details of such theoretical scheme, paying attention to the molecules and reaction/transformation processes that could be involved, with the aim to reflect on whether information processing is a key principle for their engagement into an ACS. 

### 1.3. Is There a Relationship between Chemical Evolution and Information Processing? 

One great challenge for driving interactions between ACS components toward organizational closure is how to achieve accurate recognition between them. In order to illustrate this problem it is useful to analyze how Nature deals with it in living organisms. The requisite for success of an enzyme in current cells, for instance, is not to establish correct interaction with the substrate, but rather to discriminate between similar substrates and exclude those that do not lead to a transformation process that is useful (functional) to the cell [23]. Supramolecular chemistry has extensively shown that recognition alone is efficient in the absence of noise, i.e., of similar competitive structures [24]. In molecular networks, on the contrary, discrimination between molecules seems to require a special way to control the network dynamics in which structural information of the molecules determines the network organization. In cells, this need for discrimination between competing cellular processes determines that most proteins dissipate energy to perform their functions with sufficient selectivity and specificity. This normally occurs through the action of molecular machines, as ensembles of proteins and other biomolecules that load energy into a reversible “tense” state, followed by an irreversible ratchet process that involves consumption of molecular fuels such as adenosine triphosphate (ATP), while the ensemble returns to its original “relaxed” state [25,26]. Such machines are normally able to discriminate between the correct substrate (i.e., the one that leads to a functional product and contributes to the system´s maintenance) and noise molecules that cause non-functional or malfunctioning systems. The ratchet-like discriminative process allows information to accumulate, which the system uses not only to build its own components but also to “know” how and when they must be built. Some researchers have proposed that biological molecular machines (e.g., transmembrane transporters and ribosomes) actually behave as a material implementation of Maxwell demons [27], as information-managing entities at the expense of ATP hydrolysis [23,25]. Energy dissipation in this context is the price to pay for maintaining organizational closure in living organisms, as it allows information management to ensure the constitutive cell processes are well above the constraints of noise.

The above reflections suggest that there are universal principles pervading the way information processing determines an ever-increasing dynamic complexity of the material world, particularly in (and possibly toward) its living manifestations, and raise the question of whether there is a causal relationship between information processing and evolution also in prebiotic stages. Yet, the difficulty in answering this question lies in the way biological systems process information, following machine-like operational sequences determined by the DNA software, which is not subject to extrapolation to ACSs. Biological molecular machines act as Maxwell demons because they are embedded in the complex metabolic network that supports the cell life cycle. In other words, their capacity for information processing is intimately related to the functional character of the process they carry out [28]. Information processing in ACSs could not have relied on the complex molecular machines of current biochemistry, and must have arisen from spontaneous self-organization of simple molecular components and the constraints derived from it. The potential of ACSs to preserve, through some kind of information management, processes that are favorable to their persistence, while discarding others that are detrimental, would represent an evolutionary drive in itself. This stage of evolution, prior to life and, thus, prior to the establishment of biological evolutionary mechanisms, was probably crucial in the origin of life.

## 2. Chemical Information, Meaning and Interpretation 

### 2.1. Information and Meaning at the Molecular Level 

A major problem when diving into the possible informational substrate of chemical processes that led to the first living protocells is the various misconceptions that exist about information. In particular, there is usually a misinterpretation of the duality between content and meaning of information, and despite being a central concept across science, is employed differently in the different scientific disciplines. Engineering and the natural sciences (communication engineering, computational theory and quantum physics) place the focus on *syntactic information*, informational content understood as an abstract/mathematical magnitude. Cognitive and social sciences, on the other hand, emphasize *semantic information* (what information refers to) and *pragmatic information* (information that was not known by the receiver), and thus it is not redundant [29,30]. With such multiple notions of information, it is difficult to provide insight into the physical distinctness of informational processes compared to other kinds of physical relationships. Great efforts have been made to build up a coherent view of biological information and its role in evolution [31], embracing both the syntactic nature of genetic information, contained in a digitally-coded molecular platform (DNA) [32], and the semiotic character of processes by which genes specify biological forms and functions (i.e., biosemiosis) [33]. The question is where this intricate relationship between the syntactic and semantic dimensions of biological information originates. 

When dealing only with syntactic information, the signal carrier can, in principle, be of any particular nature [29,30]. In the physicochemical context, the reactions used to quantify an analyte in an unknown material and the electromagnetic radiation that passes through a given sample in any spectroscopic experiment are examples of processes that convey information, yet only in the syntactic sense, as a potential to inform, dependent on a human to interpret the signal. Shannon´s negative characterization of information as negentropy (i.e., with respect to the potential variety of configurations of the signal carrier) is useful to understand this potential to inform, but it tells nothing about how to interpret it. In Shannon´s theory of communication, information is the amount of uncertainty removed by the reception of a given signal, and so measuring information means comparing the potential variety of signal configurations with the configuration actually transmitted [34,35]. Stochastic thermodynamics have recently shown how Shannon entropy has a precise physical meaning, determining the energetics of non-equilibrium processes in systems coupled to a thermodynamic reservoir [36]. In that kind of situation, a chemical system in an improbable state would be an information carrier, as it reflects the action of prior work that perturbed the system to reduce its entropy from a more probable state. This relationship between negentropy and external work is key to understanding syntactic information. However, the signal capacity to inform is not only dependent on the carrier pattern or configuration. In the semantic sense, something else contributes to information transmission [37].

In the context of biomolecular networks, for example, linking the syntactic notion of information to physicochemical work is a necessary but not sufficient condition for information processing. In other words, any difference or alteration in entropy does not constitute by itself a referential relationship. This is because semantic information requires a process of *interpretation* to have meaning or to be a reference to something else [38]. The important question then is how the concept of interpretation, derived from a cognitive or social context, can be applied at the physicochemical level. Bernd-Olaf Küppers has extensively reflected about the ontology of semantic information in biological systems and its possible emergence from prebiotic matter, linking it to the extension of information space constituted by biological macromolecules, through random prolongation of their primary structure [39]. Such an extension increases the syntactic complexity of information carriers, which is a requisite for the nucleation and evolution of semantic information. According to Küppers, information processing in biochemical systems is then related to the changes induced (and, thus, work exerted) by an informational molecule or molecular ensemble on an interpreting molecule or molecular ensemble, which can perform a function as a consequence of their interaction. 

The Biosemiotics School has also focused much attention on establishing a causal link between semiotic events and the emergence or implementation of functions that increase persistence of a living system or other kinds of autonomous agents (e.g., cybernetics). In their view, such causal link sets the basis of interpretation as a natural process [33,40]. An example of this would be the work exerted by a substrate molecule onto an enzyme upon binding, in the form of structural changes in the active site, which assist the function of catalytically transforming the substrate into a product useful to the cell. The example is only valid when the enzymatic function is embedded in the cellular metabolism, and cannot be applied to the same reaction being performed isolated in a test tube. In any case, the substrate-enzyme interaction has been informed throughout evolution by natural selection, which is not an assumption that can be taken for granted when extending the semiotic scheme to chemical evolution. A major condition for this attempt to naturalize the notion of semiosis, as a process connecting signaling and interpreting molecules/ensembles through physicochemical work, is that only out-of-equilibrium systems can perform work. The problem of information processing in prebiotic systems thus seems related to management of energetic flows, through populations of some kind of molecular sign users. 

### 2.2. The Bases of Physicochemical Semiosis

Based on the above considerations, in this and the next sections I propose an extension of the biosemiotics perspective to a specific type of chemical system, presenting a primitive form of organizational closure but without the need to fulfil the central dogma of biology. The principle by which entropy changes, induced by physicochemical work, can be subject to interpretation, is theoretically applicable to chemical systems if they fulfil a number of essential features. These include that the informational relationship must occur between molecular components within the system, which must be open, embedded in the environment, and maintain its dynamic internal processes out-of-equilibrium. Such conditioning factors restrict the type of chemical systems that could process information. The persistence of a chemical system in a non-equilibrium state capable of performing work driven by a source of information entails a very specific matching of the system´s internal dynamic organization (i.e., the network of reactions that enables self-sustainment) with extrinsic supportive environmental conditions (e.g., a source of free energy and raw materials). For such matching, the system also requires a unit identity, at least in a loose sense (e.g., through the establishment of a membrane compartment). Overall, these characteristics correspond to those of ACSs, as described in Section 1.2.

**Figure 1:**
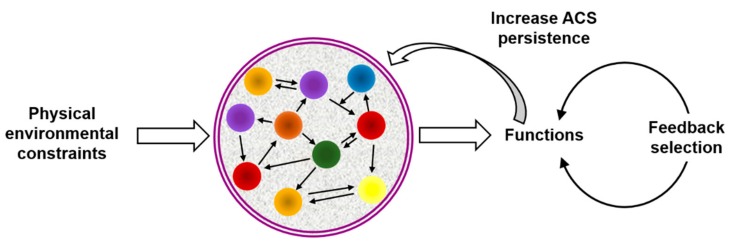
Scheme illustrating the principles underlying physicochemical semiosis in ACSs. Chemical evolution in this context would occur through a continuous correlation between environmental physical constraints and functions derived from the ACS molecular network, by which the functions can be selected and evolved through iterations of the depicted cycle. The ACS molecular network is schematically represented as a set of different molecules (colored spheres) connected through physicochemical interactions (represented by arrows) of different kinds (e.g., covalent and non-covalent transformation processes). The boundary (e.g., a membrane) must be constituted by members of the same network.

Figure 1 shows a simplified scheme of the basic principles that, according to this view, govern information processing in ACSs. The scheme aims to illustrate that correlating the environmental physical constraints with functions derived from the ACS molecular network determines an evolutionary process. The only requisite for this is that the ACS presents organizational closure, through physicochemical interactions (represented by black arrows) between the system components (represented by colored spheres), ensuring self-sustainment and reproduction of the system as a whole. In this scenario, when the interaction between two (or more) components manifests a functional advantage to the higher-level ACS state, i.e., when it leads to an improvement of its persistence by means of kinetic, spatial, energetic, or variability control, the proposed scheme implies that: (1) The ACS remains out of equilibrium, dissipating energy via interaction between the sub-ACS components. (2) A referential relationship is established through physicochemical work exerted from the informational component(s) to the interpreting component(s), which results in the realization of a function. (3) The functions executed by this kind of informational events are subject to a process of selection, based on their contribution to increase the system´s persistence.

According to the scheme in Figure 1, the ACS´s intrinsic dependence on specific external constraints (e.g., environmental conditions) links the system´s non-equilibrium dynamics to a pragmatic conception of information, i.e., to the emergence of functions that increase its persistence. The ACS autonomy in this context not only results from autocatalytic events, but also from functions that contribute to the system´s self-sustainment and reproduction. Any effective physicochemical interaction leading to a positive correlation of the ACS with the environmental conditions will tend to persist. Such correlation allows selection to operate over the functional outcome of the different informational interactions, and renders function and information as co-emerging primitive properties of ACSs, which enable transition to ever-increasing levels of dynamic complexity. Importantly, because the functions derived from information processing are causally linked to the interactions that originated them, their match defines a mechanism of *semiotic causation* [33], which has been identified in living organisms but may be seeded at the physicochemical level [41].

## 3. Chemosemiosis: Chemical Evolution Arising from an Informational Background

### 3.1. The Interest of Chemosemiotic Models

The previous section led to a semiotic perspective of ACSs, understood as assemblies of molecules that collectively interact with their environment, in a way that depends on the set of dynamic information-driven physicochemical interactions imprinted between their molecular constituents. This conclusion points to the need of a *chemosemiotic theory* to investigate the generation and processing of signs in chemical systems that are not alive but in the way to. The concept of *chemosemiosis* was coined in 1994 by Claus Emmeche [42], referring to the study of chemical signs in systems whose organization does not involve the digital/analog duality characteristic of living cells, i.e., the duality between template molecules with discrete sequence information (genetic molecules) and an analogic mode of continuous cellular dynamics in which structural information is implicit in the organization of a network of catalytic reactions (the metabolism) [43]. In Emmech´s view, however, the interpretation of chemical signs (i.e., of informational molecules or changes in their concentration) would not involve autonomous self-reproducing systems. This assumption seems very problematic, because without organizational closure ensuring autonomy there cannot be a self-referential capacity or, in other words, the possibility that a specific physicochemical event makes a difference for the chemical system´s persistence. The concept of chemosemiosis here supported is therefore narrower, and refers to heterogeneous chemical assemblies that lack template molecules with discrete sequence information but are still able to self-sustain and reproduce. To this end, autocatalysis is not the only requirement, as the autocatalytic system must be separated from the environment through a permeable boundary and be able to gather energy and feed molecules.

**Figure 2:**
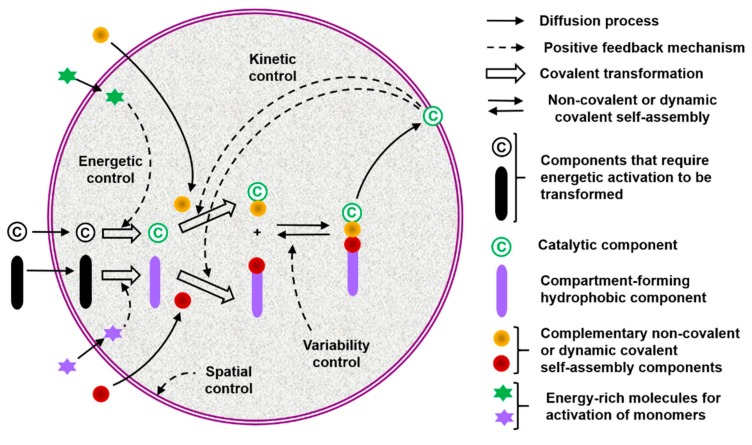
Tentative chemosemiotic model, comprising the most basic components and physicochemical interactions that would allow a protocell-like chemical system to achieve efficient kinetic, spatial, energetic, and variability control on its dynamic correlation with the environment, providing it with autonomy and evolutionary capacity.

On these bases, the qualitative and eventually quantitative analysis of a protocell performance (e.g., persistence and degree of aliveness) as a function of its semiotic activity would lead to what I call a chemosemiotic model. Protocell and chemosemiotic models are obviously related, but their perspective and utility should be different. The former focus on the components that constitute the protocell and their structural organization, while the latter will put the emphasis on the non-linear dynamics of the physicochemical interactions connecting those components. Although the limits between both views is necessarily diffuse, and there are already some research lines in systems chemistry that study non-linear, out-of-equilibrium behaviors in complex chemical systems, completing such a change of perspective will be useful to this research community. For example, it will contribute to recognize information processing as a key factor in the design of experiments toward bottom-up protocells. Consequently, Figure 2 depicts a tentative chemosemiotic model based on specific current knowledge of prebiotic systems chemistry [2]. One of the purposes of this model is to help me revising in the next subsection the molecular mechanisms that could be considered from an informational point of view in protocell research. Moreover, for this and any other possible chemosemiotic models, their main role and utility will be as theoretical tools for analysis, to help in the evaluation of the semiotic character of a given protocell configuration (see Section 4 for further details) and, therefore, on their degree of aliveness and evolutionary potential.

The model in Figure 2 comprises the most basic constituents and physicochemical interactions that would allow a protocell-like chemical system to achieve efficient kinetic, spatial, energetic, and variability control on its dynamic correlation with the environment, providing it with autonomy and evolutionary capacities. The main functional component within this model is a catalytic and amphiphilic (supramolecular or dynamic covalent) dimeric species that could form a membrane compartment and catalyze the production of the two monomers that constitute each dimer, thus achieving spatial and kinetic control, respectively. High specificity of the interaction between the two complementary self-assembling components of this functional dimer is necessary to discriminate from other possible interactions, which would lead to different non-functional species. Specific non-covalent interactions (such as hydrogen bonding) between complementary nucleobase pairs is probably the preferred way to achieve such a goal. Additionally, energetic control (activation) of the catalytic and compartment-forming hydrophobic components from inactive precursors may be necessary. These precursors and energy-rich activating molecules could be incorporated from the environment, through diffusion processes across the compartment membrane and within the ACS. In early stages of the ACS development, the functional components could also come from outside the system, but the eventual implementation of their internal synthesis would increase the persistence of the incipient protometabolic network. Importantly, structurally simple building blocks could constitute the essential components of the proposed chemosemiotic model (Table 1). The physicochemical mechanisms depicted in Table 2, in turn, can be used to explain how non-linear dynamics could develop from informational interactions between such molecular constituents. The existing literature suggests the prebiotic plausibility of each single process depicted in Figure 2 [2,3,44], while the next section details the types of mechanisms through which they could be integrated.

### 3.2. Molecular Mechanisms of Information Processing in ACSs

In the literature, it is not difficult to find mineral surfaces, organic molecules and supramolecular assemblies that could have played catalytic roles, constituted protocell membranes, acted as energy currency, or ensured selective recognition events on the prebiotic Earth. Some mineral surfaces catalyze redox and condensation reactions [45]. Various amino acids alone are able to perform acidic (His, Asp, Glu), basic (His, Lys, Arg) or nucleophilic (Asp, Glu, Cys, Lys) catalysis [46], while the capacity of some di- and tripeptides to catalyze critical biochemical transformations (e.g., aldol reactions, Michael additions, formation of phosphodiester and amide bonds, etc.) in aqueous medium and mild conditions is well known (Table 1, entry 1) [47]. Many simple, prebiotically plausible surfactant molecules are known to form micelles, vesicles, or coacervates that could host dynamic reaction networks (Table 1, entry 2) [48]. To carry out endergonic reactions, multiple activating reagents and activated monomers of nucleic acids and peptides are simple enough to be compatible with plausible prebiotic conditions, potentially enabling the coupling of endergonic and exergonic processes in an ACS (Table 1, entry 3) [2,49,50]. Finally, in order to ensure selective, non-covalent interactions between ACS components, the most logical approach would involve incorporation of canonical and non-canonical nucleobases in the structure of functional molecules [51], preferably in short sequences (2-3 nucleobases) and in cooperation with other non-covalent interactions such as van der Waals and hydrophobic forces in amphiphilic molecules, π-π stacking, metal-ligand coordination, etc. (Table 1, entry 4).

Therefore, it seems that the ingredients to fulfill the basic functionalities of an ACS need not be complex from a structural perspective, and were probably available in significant amounts on the primitive Earth. All these building blocks would certainly have been less efficient than current phospholipids, proteins and nucleic acids in performing their cellular roles, but could have humbly done the job, albeit in a more rudimentary way. The problem is to infer how they could have integrated with coherent operational dynamics in the absence of the current biochemical machinery. Learning again from the bases of biological autonomy [22], in the absence of some regulation mechanism, loading a self-reproducing vesicle with catalysts that support a simple protometabolic network in its interior would easily lead to uncontrolled autocatalytic behavior and possible instabilities due to osmotic pressure. Regulation mechanisms are also needed to control the spatial distribution of functional molecules and substrate/energy resources within the cell and across its membrane boundaries, in order to coordinate their functional engagement. The boundary itself constitutes a source of control over the exchange of energy and matter with the environment, and limits diffusion and dilution of components, all of which contributes to achieving robust maintenance of autonomy. In order to implement such kind of self-regulation, ACSs would need to accurately generate and maintain those local and global constraints (or control mechanisms), allowing the involved chemical processes to occur in proper operational sequences that are coordinated in both space and time [19]. Information processing must be considered in this respect.

Autocatalysis constitutes for example a core type of mechanisms for variability control. There are different forms of autocatalysis in which molecules can be involved [52], such as autocatalytic reactions (Table 2, entry 1) [53], autocatalytic cycles [54,55], either alone (Table 2, entry 2) or stoichiometrically coupled (Table 2, entry 3), and autocatalytic sets (Table 2, entry 4) [56]. All of these are probably critical to limit the combinatorial explosion that can be expected when each species of a mixture (both substrates and products) can react with each other in more than one possible way (e.g., the formose reaction or HCN polymerization processes). Such a combinatorial explosion is be detrimental to the accumulation of key molecular species. The operation of autocatalytic mechanisms, however, could drive the transformation of the ACS substrates toward a limited number of amplified functional molecules that engage in these autocatalytic events. They could then assist with the emergence and persistence of ACS organizational closure. This is indeed closely related to Kauffman´s notion of collective autocatalysis [57], and has an implicit computational character. Imagine for instance a catalyst node in one of these collective autocatalytic sets, carrying out a structural pattern matching to bind a substrate and execute the operation of transforming it into product(s). This has clear parallels to rewriting systems in computational theory but, as for the biomolecular machines that were described in Section 1.2, there are also significant differences. Operation in collective autocatalytic networks is stochastic rather than deterministic, and it is also reflexive, in the sense that all molecules can act as both “rules” (i.e., the catalytic function) and “computed data” (i.e., as substrates and products) [58].

In any case, the variability control through information processing that autocatalysis would confer to an ACS must be complemented by informational interactions with other elements. These elements would ensure kinetic control (e.g., adjusting the rate of a given step of an autocatalytic process), energetic control (by activating some of the species involved), and spatial control (by confining the system to prevent dilution and ensuring availability of process substrates through diffusion, crowding and transport across membranes). Regarding kinetic control, for example, one can think of small molecule cofactors that assist the incipient protometabolic network regulating the activity of certain catalysts through competitive or allosteric inhibition processes. The activity of catalysts could also be regulated by incorporation into the hydrophobic region of the ACS membrane (Table 2, entry 5) [59,60]. If the physicochemical interaction associating the catalyst with the cofactor or the hydrophobic membrane contributes to increase the robustness of self-maintenance of the ACS molecular network (e.g., by better adjusting the rate of the catalyzed reaction to the global non-equilibrium dynamics), that interaction should persist thanks to the ACS organizational closure. This implies information processing, in the sense that the physicochemical interaction (the specific non-covalent or dynamic covalent recognition process) between the catalyst and the cofactor or membrane results in a positive functional outcome (e.g., a better match of the reaction rate with the network dynamics), which discriminates against other possible configurations of the catalyst. Of course, such discrimination does not operate for a single ACS unit, but rather at the population level.

An ACS also needs to control its boundary conditions, which is achieved by compartmentalization (i.e., spatial control). Otherwise, the ACS molecular network would be directly exposed to any change in the ambient conditions, making its non-equilibrium dynamics too fragile. The compartments are normally considered to be vesicles with an internal aqueous core, but simpler two-phase self-assembled systems like droplets or coacervates could have played a role in early stages of prebiotic evolution [61]. The inclusion of the compartment component(s) in the ACS protometabolic network, supporting their formation through establishment of selective interactions that ensure their self-assembly, would let the boundary be produced internally [62]. However, even if such behavior led to compartment self-reproduction through physical autocatalysis (Table 2, entry 6) [63], it would not be a sufficient condition for the ACS to become autonomous. The compartment must also play an active role in maintaining correct osmotic pressure in the lumen and regulate the exchange of matter and energy with the environment. In current cells, these labors are performed by complex protein-based multicomponent pumps integrated in cellular membranes [64]. In prebiotic ACSs, the role of these pumps could have been played by simple peptides, coupling protocell dynamics to osmotic gradients across the boundary, which is needed for active transport and energy transduction mechanisms. The interaction of these peptides/complexes with the membrane components would be informational, as the positive correlation of their functional outcome with the environment would be selected in ACS populations with different performances. Examples of this include the osmotic gradient between the ACS interior and the environment [12,65], enabling transport processes or endergonic reactions that increment ACS persistence (Table 2, entry 7), as they could provide a key node species in the protometabolic network. Endergonic-exergonic couplings assisted by energy-rich molecules and activated monomers would also lie within this category (Table 2, entry 8) [2], as well as energy dissipating compartment self-assembly processes (Table 2, entry 9) [66].

## 4. Discussion

In sum, there are multiple ways in which informational interactions could reinforce the self-sustainment and reproduction of an ACS. I have revised only a few of them, those which have stronger experimental support. One could imagine other types of physicochemical interaction that match the proposed chemosemiotic scheme. In any case, the important conclusion from the suggested model is that chemical evolution can be conceived as a process of matter complexification with intermediate stages, where the gradually increasing degree of functional order is preserved through physicochemical interactions with a semiotic character. This semiotic nature is possible in populations of self-maintaining, compartmentalized molecular networks that present organizational closure, even if they do not possess a digital molecular platform to code information. It is not known how simple or difficult it might have been to establish such kind of systems on prebiotic Earth, but the most recent developments in prebiotic systems chemistry seem to indicate that very simple molecules can perform the basic functionalities required for an ACS to be viable (Table 1). There are also plenty of mechanisms by which these components can engage in complex non-linear dynamics (Table 2).

The power of this chemosemiotic theory would not be evident, however, if it cannot be confronted with experimental and computational protocell models, using it to explain or even predict their persistence in different scenarios. The idea is that for any kind of protocell, no matter how simple or complex is (e.g., a membrane vesicle that contains an autocatalytic macromolecule or an autocatalytic cycle, an autocatalytic network where one of the components forms vesicles or coacervates, a self-reproducing vesicle, a self-reproducing vesicle with membrane peptides that modulate permeability, or more complex alternatives), it would in principle be possible to study the extent by which the involved physicochemical interactions contribute to the protocell performance. This implies evaluating both thermodynamic and dynamic kinetic stability [9], which for the first example would be affected by confinement effects into the efficiency of the autocatalytic process and stability changes induced in the vesicle by the autocatalyst, among others. Interestingly, using non-equilibrium statistical physics to analyze semantic information from a thermodynamic point of view, Kolchinsky and Wolper have developed a computational approach to evaluate the content of semantic information in different physical systems [67]. This kind of computation could lead to practical analysis and comparison of chemosemiotic models, correlating persistence in different conditions as a function of currently vague concepts such as “value of information”, “semantic content”, and “agency”. The results of such analyses will certainly help guiding protocell research.

From a more conceptual perspective, it is important to note that the notion of chemosemiosis partly derives from inspection of the main features of information processing in living systems. Biosemiotics has already pointed out the semiotic nature of biological information and the historical character of information-driven evolution. In particular, the space of possible configurations of a species is vastly larger than its actual state, precluding evolution to fall into a state of statistical probability, which rules out its being dictated by deterministic laws [68]. Thus, if the characteristic organizational complexity of life is not the inevitable consequence of predictable lawfulness, other principles must be involved in its emergence. The principle of natural selection helps to explain the wide adaptability of biological organisms [69], but it says nothing about their origin. The concept of *aboutness*, coined by Clayton and Kauffman [70], accounts for the never-ending chain of correlations between living systems and their conditions of life, but it again refers to systems that reproduce with heritable information, which implies a great level of chemical complexity that cannot be taken for granted when discussing prebiotic stages of the pathway to life. Thus, something is missing in the current picture of life abiogenesis. The theoretical framework outlined here is devoted to decipher such a missing link that exists between chemistry and biology, which requires connecting the dynamics of chemical interaction patterns with the dynamics of signification. Proposing that the physicochemical processes constituting ACSs present a semiotic character can also help to unify the two dominant views of chemical evolution, seen as either a natural process of self-organization [71] or as a relational-constructive problem [21]. With the aim to make both views more coherent, I strongly argue that information processing should be placed at a central position in any effort to explain chemical evolution, not only in late stages but right from the simplest building blocks, which are not able to code information in a genetic manner but can functionally engage in ACSs.

## Figures and Tables

**Table 1:** Set of molecular components that could have provided the basic functions in ACSs.

Basic Function	Structure of Functional Molecular Components	
Kinetic control (catalysis)	Mineral surfaces **Oxides:** SiO_2_, TiO_2_, Al_2_O_3_ **Hydroxides:** Al(OH)_3_, Fe(OH)_3_ **Iron sulfides:** FeS, FeS_2_ **Clays:** montomorillonite, illite, bentonite, saponite, kaolinite **Phosphates:** hydroxyapatite	Amino acids **Acid catalysis:** His, Asp, Glu **Basic catalysis:** His, Arg, Lys **Nucleophilic catalysis:** Asp, Glu, Cys, Lys Di- and tripeptides **Aldol reactions:** Pro-based dipeptides (e.g., Pro-Gly, Pro-Phe, Pro-Val) **Amide and phosphodiester bond formation:** His-based di- and tripeptides (e.g., Ser-His, Ser-His-Gly, Ser-His-Asp)
Spatial control (compartmentalization)	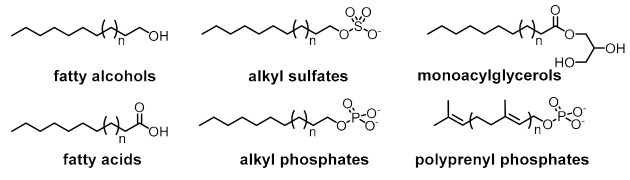	
Energetic control (to favor endergonic reactions of interest)	Activating reagents 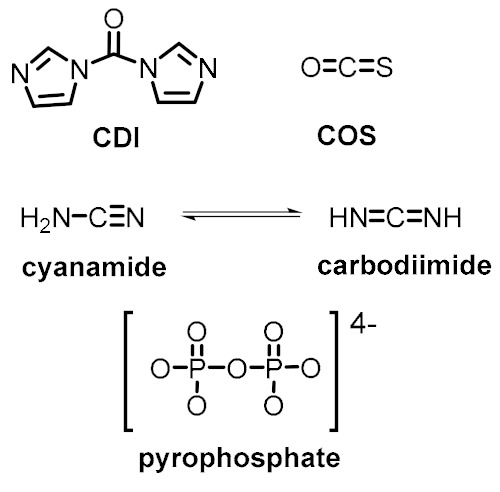	Activated monomers 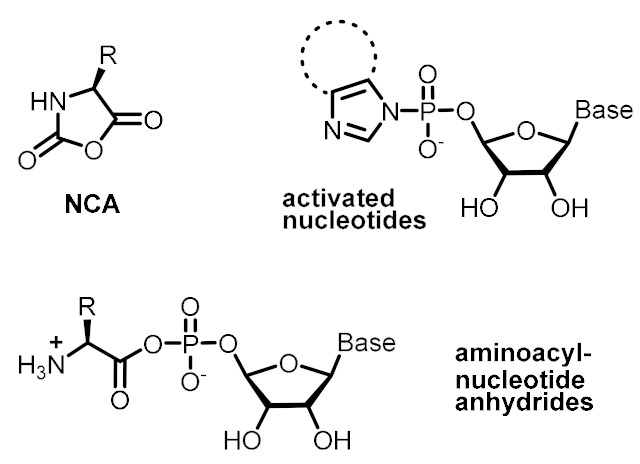
Variability control (to achieve accurate recognition between functional components)	Canonical nucleobase pairing Alternative nucleobase pairing 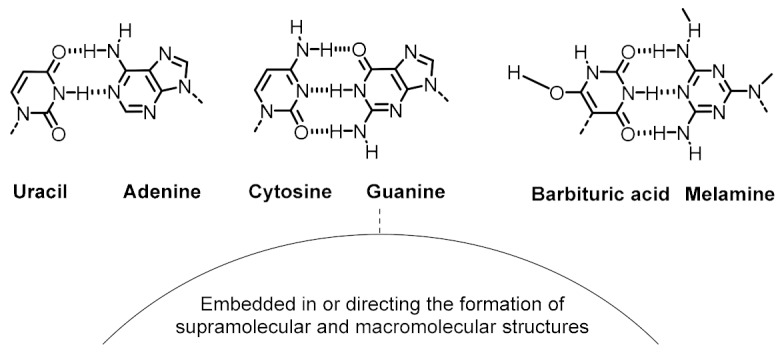	

**Table 2:** Control mechanisms to establish the basic functions of an ACS.

Control Mechanism	Functional Process
Entry 1: Autocatalytic reactions	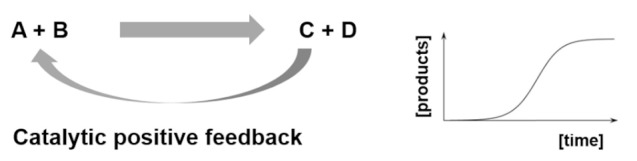
Entry 2: Autocatalytic cycles	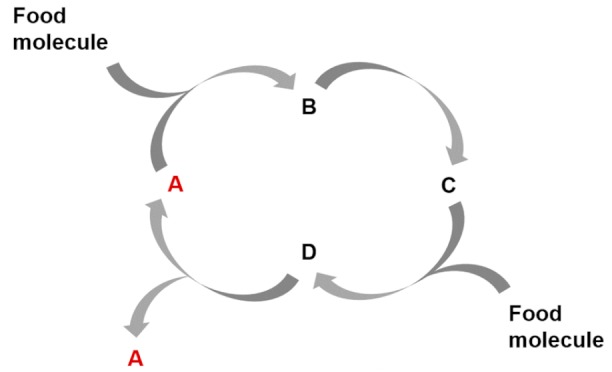
Entry 3: Stoichiometric couplings of autocatalytic cycles	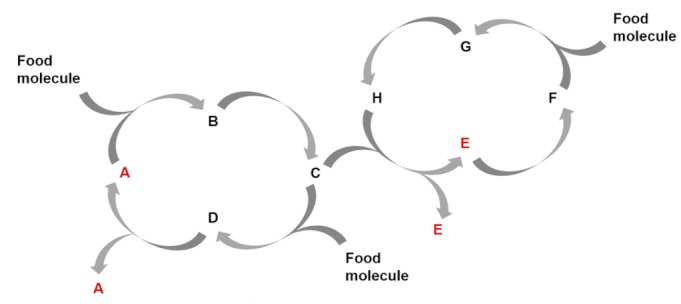
Entry 4: Catalysis mediated autocatalytic sets	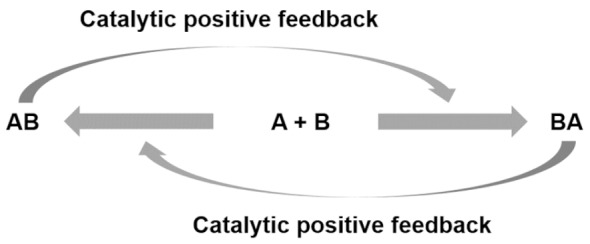
Entry 5: Catalysis in compartments	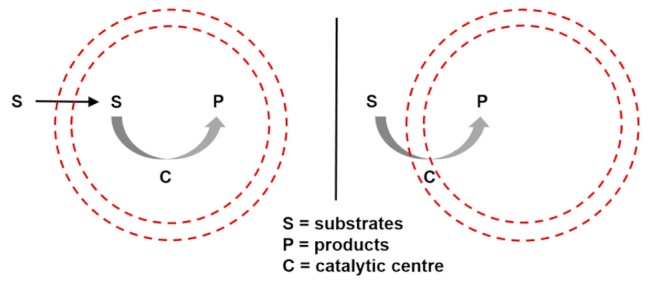
Entry 6: Compartment self-reproduction	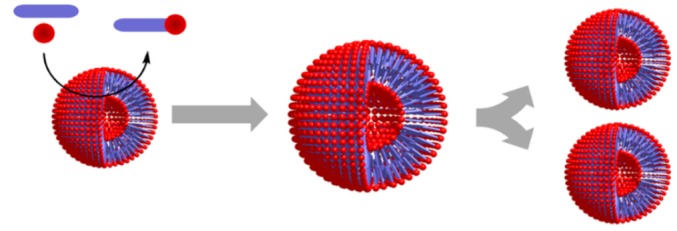
Entry 7: Osmotic couplings in compartments	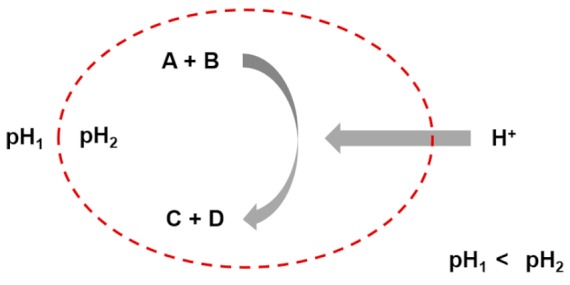
Entry 8: Endergonic – exergonic couplings	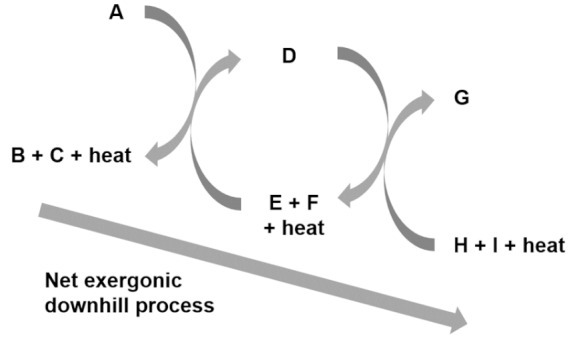
Entry 9: Energy dissipation by self-assembly	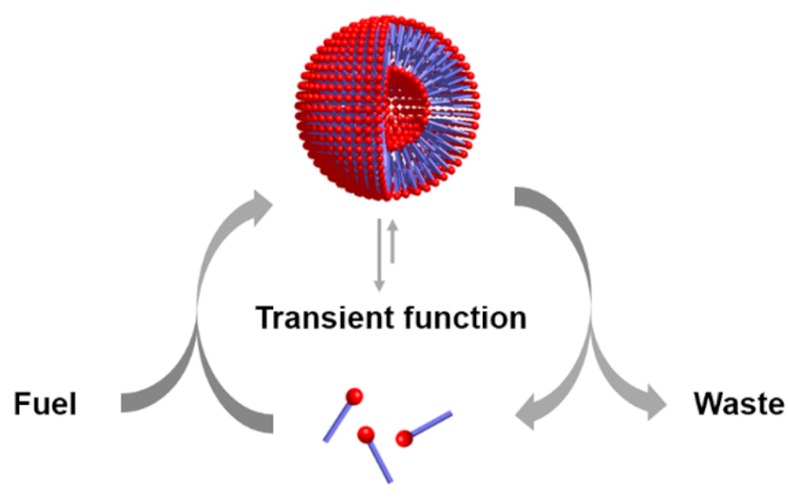

A, B, C, D, E, F, G, H, and I, are chemical species acting as substrate, intermediate or final products in the different depicted processes.
